# Supplementary material for: Cervical cancer screening in Brazilian Amazon Indigenous women: Towards the intensification of public policies for prevention
Source: PLoS One. 2023 Dec 8;18(12):e0294956. doi: 10.1371/journal.pone.0294956 (PMC10707695; doi:10.1371/journal.pone.0294956)
Supplement: S1 Table — (DOCX) [file pone.0294956.s001.docx]

| Supplemental table 1. Description of health expeditions with screening activities in indigenous women from the Brazilian Amazon from 2007 to 2019. | | | |
| --- | --- | --- | --- |
| Year | **Location/State** | **Ethnicity** | **Women screened (n)** |
| 2007 | Tapajós/PA | Riversides | 167 |
| 2008 | Tabatinga/AM | Ticunas | 92 |
| 2009 | Parintins/AM | Saterê Mauê | 83 |
| 2010 | Parintins/AM | Saterê Mauê | 93 |
| 2010 | Médio Rio Negro/AM | Baré, Tucanos, Baniwa, Yanomamis | 116 |
| 2011 | São Felix do Xingu/PA | Kayapós | 128 |
| 2011 | Jacareacanga/PA | Mundurukus | 160 |
| 2012 | São Gabriel da Cachoeira/AM | Tucanos, Tarianos, Desanos, Tuiucas, Rupdas | 96 |
| 2012 | Raposa Serra do Sol/RR | Macuxis, Wapishanas, Yanomamis | 163 |
| 2013 | Tabatinga/AM | Ticuna, Kokama, Kaixana, Kambeba, MakuYuhupi, Witota, Marubo, Canamari, Matis, Mayruna | 136 |
| 2013 | Surucucú/RR | Yanomamis | 143 |
| 2013 | Raposa Serra do Sol/RR | Macuxis, Wapishanas, Yanomamis | 50 |
| 2014 | Xingú/MT | Macuxis, Yanomamis, Wai-Wai, Taurepang, Wapishana, Patanoma, Ingaricó, Wecuena, Sanomã | 54 |
| 2014 | São Pedro/MT | Xavantes | 185 |
| 2015 | Feijó/AM | Ticunas and neighbors | 128 |
| 2015 | Normandia/RR | Yanomamis | 168 |
| 2015 | Jacareacanga/PA | Mundurukus, Kaiapós, Apiacás, Kayabi | 156 |
| 2016 | Parintins/AM | Saterê Mauê – Hixcariana | 123 |
| 2016 | Assunção do Içana/AM | Baniwa, Baré, Curupaco, Desana | 135 |
| 2017 | Iauaretê and Paricachoeira /AM | Tucanos, Dessanos, Tarianos, Pipatapuia, Tuyucas, Rupdas, Shanenawa, Kulina, Kampa, Kaxinawá, Nukini, Poyanawa | 250 |
| 2017 | Feijó/AC | Yaminawa, Arara, Katukina, Yanawá | 193 |
| 2018 | Lábrea/M | Apurinã, Paumari, Jarawara, Jamandi, Suruwaha, Kokama | 184 |
| 2018 | Montes Altos/MA | Guajajara, Gavião, Awaquajá, Guajá, Urubukapor, Krikati, Timbira | 216 |
| 2019 | Santa Isabel do Rio Negro/AM | Desana, Baré, Piratupuia, Tucano, Siriano, Baniwa, Yanomami | 173 |
| 2019 | São Miguel de Arapiuns/PA | Riversides and indígenous | 121 |
